# Primary iliopsoas abscess due to *Staphylococcus aureus* bacteremia

**DOI:** 10.1590/0037-8682-0264-2020

**Published:** 2020-11-13

**Authors:** Chee Yik Chang, Sing Chiek Teoh

**Affiliations:** 1 Sarawak General Hospital, Medical Department, Kuching, Malaysia.

**FIGURE 1:**
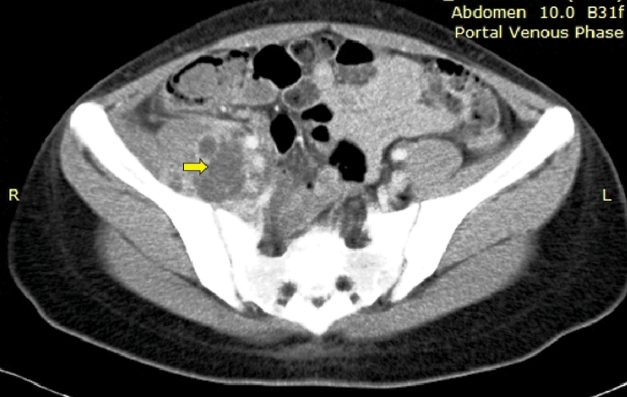
Computed tomography (CT) of the abdomen and pelvis shows multiloculated rim-enhancing collections at the right iliopsoas muscle

A 17-year-old girl with poorly controlled type I diabetes mellitus presented with a 2-week history of fever and limping. She reported no history of previous trauma. On examination, there was reduced range of motion in the right hip and a positive psoas sign. The cardiovascular and lung examinations were unremarkable. Blood investigations showed leukocytosis (25 x 10^3^/uL). The chest radiograph and transthoracic echocardiography findings were normal. Because psoas abscess was suspected, CT of the abdomen and pelvis was performed, which revealed multiloculated rim-enhancing collection at the right iliopsoas muscle (Figure 1). She then underwent percutaneous drainage in which a copious amount of purulent material was drained. The blood culture revealed *Staphylococcus aureus* (sensitive to oxacillin and trimethoprim-sulfamethoxazole). In the ward, she was treated with intravenous cloxacillin 2 g every 4 hours for 2 weeks. One week later, the musculoskeletal ultrasonography showed a reduction in the size of the abscess. Her fever subsided, and the right hip pain started improving. Subsequently, she took oral trimethoprim-sulfamethoxazole for another 4 weeks, which had resulted in ultrasonographic resolution of the right iliopsoas abscess. 

Iliopsoas abscess can be classified as a primary or secondary abscess. The former occurs as a result of hematogenous spread of an infectious process from an occult source, while the latter occurs when there is a direct spread of infection from an adjacent structure^1^. The presenting features of iliopsoas abscess were non-specific, and the classic Mynter’s triad of fever, pain, and limping is present in 30% of the patients^1^ ^,^ ^2^. *S. aureus* and *Escherichia coli* are the most common causative organisms of primary and secondary iliopsoas abscesses, respectively^3^. Treatment of iliopsoas abscess involves the use of appropriate antibiotics along with abscess drainage^1^. Iliopsoas abscess is a rare but serious complication of *S. aureus* bacteremia and should be suspected in patients presenting with fever and limping. Early diagnosis and prompt treatment are crucial to a successful outcome. 
